# Robotic Total Mesorectal Excision for Rectal Cancer: Short-Term Oncological Outcomes of Initial 178 Cases

**DOI:** 10.1007/s13193-020-01212-5

**Published:** 2020-09-08

**Authors:** C. Ramachandra, Pavan Sugoor, Uday Karjol, Ravi Arjunan, Syed Altaf, C. Srinivas, B. V. Prakash, Vijay Patil

**Affiliations:** Department of Surgical Oncology, Kidwai Memorial Institute of Oncology, Bengaluru, Karnataka India

**Keywords:** Rectal cancer, Robotic rectal resection, Robotic total mesorectal excision, Da Vinci surgical system

## Abstract

Emerging techniques in minimally invasive rectal resection include robotic total mesorectal excision (R-TME). The Da Vinci Surgical System offers precise dissection in narrow and deep confined spaces and is gaining increasing acceptance during recent times. The aim of this study is to analyse our initial experience of R-TME with Da Vinci Xi platform in terms of perioperative and oncological outcomes in the context of data from recently published randomised ROLARR trial amongst minimally invasive novice surgeons. Patients who underwent R-TME or tumour specific mesorectal excision for rectal cancer between May 2016 and November 2019 were identified from a prospectively maintained single institution colorectal database. Demographic, clinical-pathological and short-term oncological outcomes were analysed. Of the 178 patients, 117 (65.7%) and 31 (17.4%) patients had lower and mid third rectal cancer. Most of the tumours were locally advanced, cT3–T4: 138 (77.5%). One hundred/178 (56.2%) underwent sphincter preserving TME. Eighty-seven (48.8%) were grade II adenocarcinoma. Nonmucinous adenocarcinoma was the predominant histology, 138 (78.4%). One hundred one cases (56.7%) were pT3. The mean number of lymph node yield was 13 ± 5. Distal resection margin and circumferential resection margin were positive in 2 (1.12%), 12 cases (6.74%) respectively. Eleven cases (6.7%) had to be converted to open TME. Mean blood loss and duration of surgery was 170 ± 60 ml and 286 ± 45 min respectively. Five percent cases had an anastomotic leak. Grade IIIa–IIIb Clavien Dindo (CD) morbidity score was reported to be in 12 (6.75%) and 10 (5.61%) cases. Median length of hospitalisation was 7 days (range 4–14 days). Perioperative and pathologic outcomes following robotic rectal resection is associated with good short-term oncological outcomes and is safe, effective, and reproducible by a minimally invasive novice surgeon.

## Introduction

Neoadjuvant chemoradiotherapy (NACRT) has a major role in the treatment of locally advanced rectal tumours [1]. Oncological outcomes have improved following widespread acceptance of the principles of TME [2]. Surgical techniques govern oncological outcomes in rectal cancer surgery. Tumour-specific mesorectal excision or total mesorectal excision (TME) and achieving a negative circumferential resection margin (CRM) are associated with lower recurrence rates and improved overall survival [3–9].

There have been numerous prospective randomized studies about the superior short-term outcomes of laparoscopic surgery for rectal cancer in comparison with open rectal resections [10–14]. Few studies have raised concerns on the quality of TME, composite pathological outcomes, and the oncological safety associated with the laparoscopic total mesorectal excision (L-TME approach) [15–17]. The question remains still open after the publication of the results of the latest trials [17–20].

Poor visibility coupled with difficult access in deep and narrow pelvis makes dissection and distal division a technical challenge, which may result in suboptimal pathological outcomes and hence are the fundamental concerns with L-TME. This has led some surgeons to adopt robotic-assisted approach which aims to improve the ease and quality of TME while still retaining the potential benefits of minimally invasive approach.

Robotic-assisted approach offers magnified three dimensional vision, a surgeon-controlled camera and operating platform, instruments with various degrees of freedom and articulation, enhanced ergonomics and tremor filtration [21]; these advantages may translate to superior TME quality and improved autonomic functional outcomes [22–24].

ROLARR randomized trial, compared laparoscopic versus robotic surgery and reported robotic approach may have a lower overall conversion rate particularly benefiting obese men subgroup. The study did not find any statistical significant differences in the rest of the short-term outcomes including bladder and sexual dysfunction [25].

R-TME at our institution is being performed since 2016 and has become the preferred procedure of choice in patients who are suitable for a minimally invasive approach. The objective of this study was to prospectively evaluate the initial experience with R-TME and associated perioperative and oncologic outcomes in the context of data from recently published ROLARR trial.

## Materials and Methods

Retrospective review of a prospectively maintained colorectal database identified 178 patients who had underwent robotic rectal resections using Da Vinci Xi Robotic system (Intuitive Surgical Inc., Sunnyvale, CA, USA) for biopsy confirmed primary rectal adenocarcinoma or melanoma at Kidwai Memorial Institute of Oncology, Bengaluru, India, between May 2016 to November 2019. Rectal cancer was defined as tumours that were ≤ 15 cm from the anal verge and were grouped into upper (11–15 cm), mid (6–10 cm), and lower (≤ 5 cm) based on distance from anal verge.

### Preoperative Staging and Neoadjuvant Treatment

All patients underwent preoperative staging of pelvis with magnetic resonance imaging (MRI) and computed tomography (CT) scans of the abdomen and thorax. NACRT was offered to patients with clinical stage T3–T4 N0 or any T N+ with or without mesorectal fascia (MRF) involvement. The NACRT regimen included oral capecitabine–based chemotherapy and external beam radiation (a total dose of 50.4 Gy in 25 fractions). After completion of NACRT, all patients underwent restaging pelvic MRI. If MRF/CRM remained positive, additional 4 cycles of capecitabine- and oxaliplatin-based chemotherapy were administered. Surgery was performed between 7 and 8 weeks postNACRT.

Early T3 (T3a–T3b) [26]**,** CRM/MRF negative cases received short-course radiotherapy (SCRT: 25Gy in 5 fractions) and underwent surgery between 3 and 7 days postSCRT. All patients received adjuvant chemotherapy for a period of 6 months.

### Eligibility Criteria

Patients who underwent abdomen/pelvic dissection after docking were included in the analysis. Robotic surgery was not offered for patients requiring extended resection, complex abdominal wall reconstruction, or synchronous liver resection with laparotomy. Cases with presence of significant intraabdominal adhesions limiting access to the distal colon or pelvis or peritoneal deposits on staging/diagnostic laparoscopy were excluded.

### Outcome Assessment

The primary measures of this analysis were perioperative and pathological outcomes. < 1 mm between deepest tumour extension to the CRM was defined as positive CRM while < 1 mm between the lowest aspect of tumour and distal cut edge of specimen was considered as a positive distal resection margin (DRM) [27].

### Defining Conversion

Conversion to open surgery was defined as the use of an abdominal incision to continue the procedure under direct visualization before completion of the TME, due to any cause.

### Surgical Complications

Any adverse events within and after 30 days after surgery were defined as postoperatively early and delayed complications respectively. Anastomosis leakage was defined as feculent discharge in surgically placed drains, radiological evidence of contrast extravasation or perianastomotic collection requiring drainage, or anastomotic dehiscence as determined on digital rectal examination or flexible sigmoidoscopy.

### Technique of Totally Robotic TME for Rectal Cancer

In the present study, all patients underwent single docking, single stage and complete R-TME using the Da Vinci Surgical System Xi [28].

### Data Collection and Statistical Analysis

All demographic, operative, pathological and postoperative recovery data were obtained from the prospectively maintained colorectal database. Surgical complications were stratified by Clavien Dindo classification system [29]. All statistical analyses were carried out with the Statistical Package for the Social Sciences version 21 (SPSS Chicago, IL, USA). Continuous variables were used to derive the mean ± SD (SD, standard deviation).

## Ethics

The data of the present study were collected in the course of common clinical practice, and, accordingly, the signed informed consent was obtained from each patient for any surgical and clinical procedure. The study protocol conforms to the ethical guidelines of the World Medical Association Declaration of Helsinki: Ethical Principles for Medical Research Involving Human Subjects adopted by the 18th WMA General Assembly, Helsinki, Finland, June 1964, as revised in Tokyo 2004. No approval of the institutional review committee was needed.

## Results

**Fig. 1:**
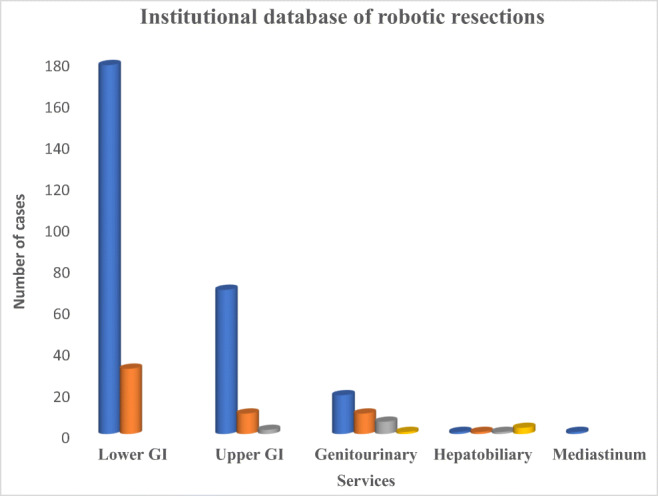
Institutional database of robotic resections

A total of 335 robotic resections were performed during the study period of which 178 cases underwent robotic rectal resection Table 1, Fig. 1. R-TME was performed initially by a single surgeon with 2 additional surgeons progressively transitioning from open to robotic during the study period with annual increase in the total number of cases performed robotically.

**Table 1:** Institutional database of robotic resections

Procedures	Numbers (*n*) = 335
1. Lower GI	210
a. R-TME	178
b. R-CME	32
2. Upper GI	82
a. TTE	70
b. Subtotal gastrectomy	10
c. Total gastrectomy	02
3. Genitourinary	36
a. Radical nephrectomy	19
b. RCIC	10
c. RARP	06
d. Adrenalectomy	01
4. Hepatobiliary	06
a. PPPD	01
b. DPS	01
c. Radical cholecystectomy	01
d. Simple cholecystectomy	03
5. Mediastinum and thorax	01
a. Thymoma excision	

*R-TME* Robotic total mesorectal excision, *R-CME* robotic complete mesocolic excision, *RCIC* radical cystectomy with ileal conduit, *RARP* robotic-assisted radical prostatectomy, *PPPD* pylorus preserving pancreatico-duodenectomy, *DPS* distal pancreatico-splenectomy

### Patient Characteristics

The demographic characteristics are summarized in Table 2. Median age was 51 years (range: 23–87 years). 51.7% (*n* = 92) and 48.3% (*n* = 86) were men and women respectively. Mean body mass index was 22.8 ± 4. Lower third and middle third rectal tumours amounted for 65.7% (*n* = 117) and 17.4% (*n* = 31) cases respectively. Locally advanced T3–T4 tumours were predominant, accounted for 77.5% (*n* = 138) of the cases. 65.5% received neoadjuvant therapy.

**Table 2:** Baseline characteristics of the patients

Variables	Numbers (*n*)
1. Median age	51 years (23–87 years)
2. Gender	
a. Male	92 (51.7%)
b. Female	86 (48.3%)
3. Mean BMI	22.8 ± 4
4. ASA	
a. I–II	18 (66.2%)
b. III–IV	60 (33.7%)
5. Tumour location	
a. Upper third	30 (16.9%)
b. Middle third	31 (17.4%)
c. Lower third	117 (65.7%)
6. Preoperative T stage	
a. T1–T2	40 (22.4%)
b. T3–T4	138 (77.5%)
7. Preoperative N stage	
a. Node negative	64 (35.9%)
b. Node positive	114 (64.0%)
8. Baseline CRM	
a. Free	142 (79.77%)
b. Involved	36 (20.24%)
9. Neoadjuvant treatment	
a. Yes	117 (65.5%)
b. No	61 (34.5%)

### Operative Outcomes

Table 3 illustrates operative parameters. One hundred sixty-seven/178 underwent complete robotic rectal resections. Sphincter preservation procedures amounted for 56% (*n* = 100) of cases, and the remaining 44% (*n* = 78) were Abdomino-perineal resections (APR). Mean total operating time, docking and surgeon console time were 286 ± 45 min, 13 ± 5 min and 220 ± 20 min respectively. Mean blood loss was 170 ± 60 ml.

**Table 3:** Operative outcomes

Variables	Numbers
1. Surgical procedure	178
a. Abdomino-perineal resection	78
b. Low anterior resection	65
c. Anterior resection	25
d. Intersphincteric resection	07
e. Posterior exenteration	03
2. Mean total duration of surgery	286 ± 45 min
3. Mean docking time	13 ± 5 min
4. Mean surgeon console time	220 ± 20 min
5. Mean blood loss	170 ± 60 ml
6. Conversion rates	11 (6.7%)

### Docking Time

**Fig. 2:**
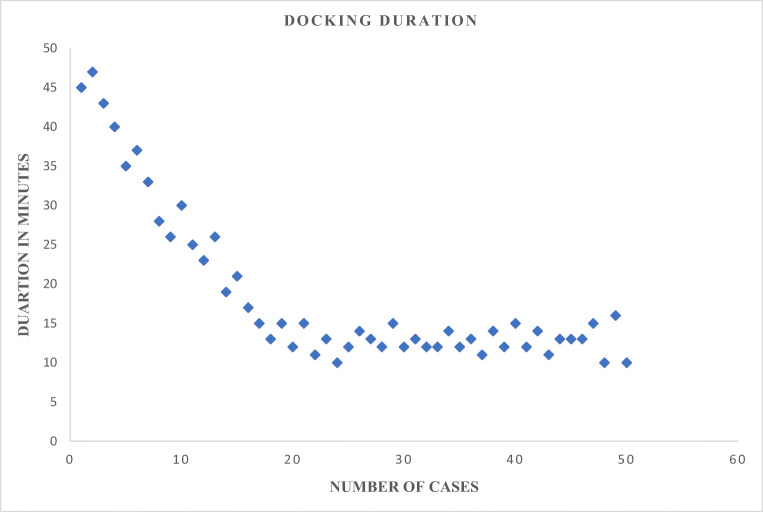
Duration of docking

Mean docking time is 13 ± 5 min. Our analysis showed that with a standardised six port technique the docking time plateaus after 18 consecutive cases (Fig. 2).

### Time Stratified Analysis

**Fig. 3:**
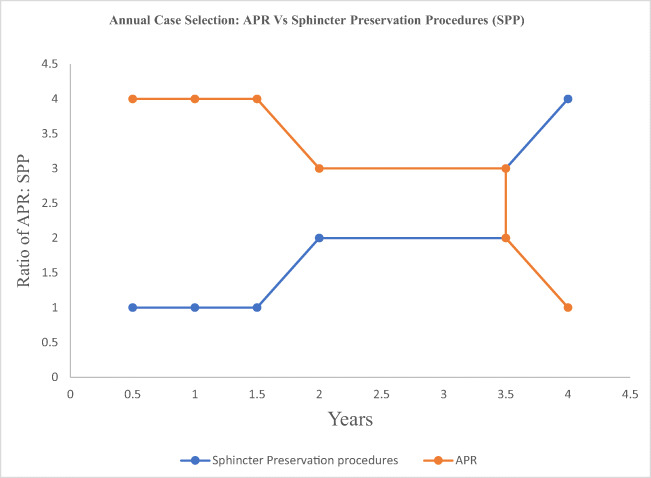
Annual case selection distribution

During the first year, the ratio of APR to sphincter preserving procedures was 4:1, with increasingly gaining experience the ratio was 2:2 during the first half of the second year and 1:4 thereafter (Fig. 3).

An annual analysis of cases showed that the mean operative time during the first year 180 ± 30 min and 230 ± 40 min and 300 ± 30 min during the second and third year.

### Conversion Rates

Eleven out of 178 had a conversion to open approach. Conversion was more often in men and in whom sphincter preservation strategy was desired. Seven out of 11 converted cases (63.63%) were amongst men and 36.37% women (*p* = 0.04) in whom a sphincter preservation was planned. Reasons for conversion are listed in Table 4.

**Table 4:** Factors favouring conversion to open approach

Unfavourable parameters	Numbers (*n* = 11)
1. Uncontrolled bleeding from Inferior mesenteric artery	01
2. Hem-o-lock clip slippage from inferior mesenteric artery stump	01
3. Common iliac artery bleeding while dissecting the left ureter	01
4. Peri-hilar bleeding while splenic flexure mobilisation	01
5. Descending colon perforation while mobilising splenic flexure	01
6. Pre-sacral venous plexus injury and bleed	01
7. Rectal perforation while dividing meso-rectum	01
8. Rectal disruption while insertion of stapler	02
9. Iatrogenic tumour perforation	01
10. Incidentally detected multiple bulky lateral pelvic lymph nodes	01

In 18.2% cases (17 of 93 sphincter preservation procedures (ISR excluded)) midline utility incision was utilised for rectal division (after completion of TME).

### Histopathologic Outcomes (Table 5)

**Fig. 4:**
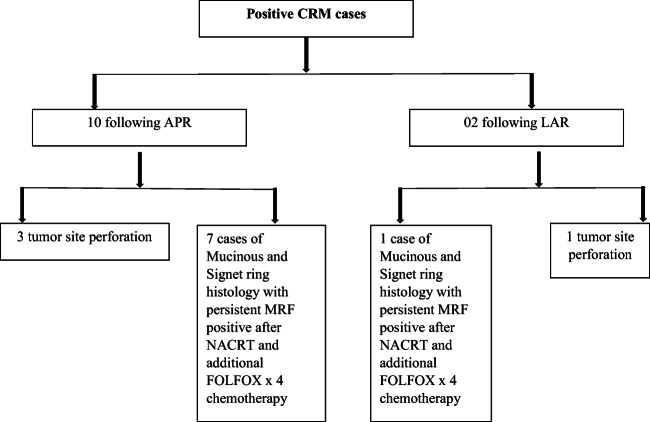
Consort of positive CRM cases

Signet cell adenocarcinoma constituted a small volume of 08 (4.5%) cases. 56.7% (*n* = 101) cases were reported to be pT3. Complete pathological response was noted in only 2.8% (*n* = 5) cases. Mean distal resection margin length was 2.6 ± 1.8 cm. Two cases (1.12%) (one low anterior resection and one intersphincteric resection) had microscopic distal resection margin involvement on final histopathological examination for which a second surgery, APR, was performed on 12th and 15th postoperative day respectively. 6.74% (*n* = 12) cases had a positive CRM, and most of these cases had underwent an APR (Fig. 4). Mean number of lymph nodal retrieval was 13 ± 5.

**Table 5:** Histopathologic outcomes

Variables	Numbers (%)
1. Histopathology	*n* = 178
a. Adenocarcinoma	138 (78.4%)
b. Mucinous adenocarcinoma	30 (17.0%)
c. Signet ring adenocarcinoma	08 (4.5%)
d. Melanoma	02 (1.12%)
2. Grade	*n* = 176
a. I	51 (28.7%)
b. II	87 (48.8%)
c. III	38 (22.4%)
3. Pathological T stage	
a. pT1	9 (5.1%)
b. pT2	63 (35.4%)
c. pT3	101 (56.7%)
d. CPR	5 (2.8%)
4. Pathological nodal stage	
a. N0	96 (53.9%)
b. N1	43 (24.1%)
c. N2	39 (21.9%)
5. Distal resection margin status	
a. Negative	176 (98.8%)
b. Positive	02 (1.12%)
6. Mean distal resection margin length	2.6 ± 1.8 cms
7. Circumferential resection margin status	
a. Negative	166 (93.25%)
b. Positive	12 (6.74%)
8. Mean number of lymph nodal yield	13 ± 5

*CPR* Complete pathological response

### Postoperative Complications

Outcomes of postoperative recovery are presented in Tables 6 and 7. Mean time to first flatus passage was 2 ± 1 days; time to resume to oral intake of liquids was 1.5 ± 0.5 day. Mean length of hospital stay was 6 ± 2 days for APR procedures and 8 ± 1 days following sphincter preserving procedures. Urinary catheter removal was performed after 6 days following sphincter preservation procedure and after 8 following APR. Eight/100 (8%) patients had anastomotic leakage. Twelve/178 (6.74%) and 10/178 (5.61%) cases had Clavien-Dindo grade IIIa and IIIb complications respectively. There was one postoperative death, due to an unexpected cardiac event that occurred on postoperative day 6 following APR.

**Table 6:** Postoperative outcomes and complications

Parameters	Numbers (%)
1. Mean time to first passage of flatus (days)	2 ± 1
2. Mean time to resume to oral intake of liquids (days)	1.5 ± 0.5
3. Mean length of hospital stay (days)	
a. APR	6 ± 2
b. Sphincter preservation R-TME	8 ± 1
4. Anastomotic leak rate	8 (8%)
5. Clavein-Dindo complications	
a. Grade I	125 (70.22%)
b. Grade II	29 (16.29%)
c. Grade IIIa	12 (6.74%)
d. Grade IIIb	10 (5.61%)
e. Grade IV	01 (0.56%)
f. Grade V	01 (0.56%)

*APR* Abdomino-perineal resection, *R-TME* robotic total mesorectal excision

**Table 7:** Interventions for anastomotic leak

Procedures	Interventions
Two cases of low AR without DS	Exploration followed by peritoneal lavage and loop ileostomy
Low AR with DS	Laparoscopic peritoneal lavage in view of minimal pelvic confined contamination
Two cases with partial coloanal anastomotic dehiscence following ISR	Drainage of the pelvic collection with trans anal repair of the partial anastomotic dehiscence site
Low rectovaginal fistula following low AR	Completion APR
Two cases of low AR with DS in hemodynamically stable patients	Radiologically guided pigtail drainage of collection

*AR* Anterior resection, *ISR* intersphincteric resection, *DS* diversion stoma, *APR* abdomino-perineal resection

## Discussion

This study is perhaps the largest series from the Indian subcontinent, and to the best of our knowledge, this is the only Indian series identified in the literature from a regional cancer centre which has analysed short-term oncological outcomes following robotic rectal resection by a robotic novice surgeon.

ALaCaRT and ACOSOG Z6051 randomised trials failed to demonstrate noninferiority of laparoscopic surgery with open surgery for rectal cancer in terms of pathological success, raising concerns about its effect on clinical outcomes. Planned analysis of secondary outcomes after a minimum follow-up of 2 years has not found significant differences in disease-free survival nor locoregional recurrence, although estimates of treatment effect favoured open resection indicating that an alternative platform such as robotics may improve oncological outcomes of minimally invasive surgery. Robotic rectal resection was developed to overcome the limitations of conventional laparoscopy and to achieve a superior quality of oncologic resection [30].

As a new surgical procedure, worldwide R-TME is adopted increasingly by surgeons. Patient selection is of utmost importance during the initial period of learning. In our series, all three surgeons were making a transition from open to robotic approach and were novice with laparoscopic approach; hence during the initial year, the surgeons selected cases suitable for robotic APR which were less complex until more experience was gained; this explains our APR rates of 43.82% (78/178) cases. During the first year, the ratio of APR to sphincter preserving procedures was 4:1; with increasingly gaining experience, the ratio was 2:2 during the first half of the second year and 1:4 thereafter.

Existing literature demonstrate longer operative time for robotics compared with open and laparoscopic rectal resections [31–34]. ROLARR trial reported a mean duration of surgery of 298.5 ± 88.71. Mean operative time in our series is 286 ± 45 min. Further, an annual analysis of cases showed that the mean operative time during the first year 180 ± 30 min and 230 ± 40 min and 300 ± 30 min during the second and third year. This progressively increasing annual operative time is due to routine splenic flexure mobilisation and more number of sphincter preservation procedures which are been performed from second year onwards.

The landmark laparoscopic rectal cancer trials, MRC CLASICC, COLOR-II, ACOSOG-Z6051, ALaCaRT reported a conversion rates of 34%, 16%, 11% and 9% respectively. The conversion rates reported in ROLARR trial is 12.2% in the laparoscopic group and 8.1% in the robotic-assisted group. The present series reports a conversion rate of 6.7% (11/178). Table 4 illustrates the reasons for conversion, and conversion rates were more often in men and when the intended procedure was sphincter preservation procedure as compared with APR. In 5/11, failure to progress into the pelvis was noted and more often in the initial few cases. Indeed, the rate of conversion in the current series is lower than reported in ROLARR but consistent with other reports from experienced centres [35]. Optimal preoperative imaging, evaluation and case selection are the attributable factors for the low conversion rates.

Robotic approach seems to facilitate mesorectal dissection, particularly in mid and low rectal tumours, hence one of the major benefit thought to be conferred by this novel approach was lower positive CRM rates. Our evaluation of CRM positivity and DRM involvement assessed the quality of mesorectal excision. Our study reports 12/178 (6.74%) CRM positive rates and 2/178 (1.12%) DRM involvement.

CRM positive was more common amongst patients who had underwent APR (Fig. 3). Ten/12 and 2/12 patients with positive CRM had underwent APR and low AR respectively. Seven/10 APR patients with positive CRM had persistent MRF involvement despite NACRT and additional FOLFOX × 4. Three/10 APR had positive CRM secondary to tumour perforation. One patient with DRM involvement following intersphincteric resection (ISR) underwent completion APR; the other ISR patient did not consent for APR and is on follow-up. Our CRM positive rates are slightly higher than ROLARR trial, reported to be 5.1%; this is probably attributed to undertaking APR procedures for mucinous or signet ring adenocarcinoma with persistently MRF positive despite NACRT and additional chemotherapy. The response to neoadjuvant treatment in this histology group is nonfavourable [36, 37].

The mean time to fist passage of flatus resume to oral liquid intake 2 ± 1 and 1.5 ± 0.5 days, respectively. Median length of hospitalisation was 7 days (range 4–14 days). Further gains in perioperative care and reduced length of stay may be possible by standardization of enhanced recovery after surgery programs. The overall complications (CD grade ≥ 2) reported in the present review is 29.76%. In the present study, major complications requiring radiologic intervention or surgical treatment were included in CD grade IIIa–IIIb. Of the 100 patients with an anastomosis, 8 (8%) cases had a leak. All the 8 cases required interventions as listed in Table 7. ROLARR trial reports overall 30-day complication and anastomotic leak rates of 33.1% and 12.2% respectively.

Although the results of this study are comparable with those of the ROLARR trial, there exist significant differences in the case selection and types of procedures between these two studies.

Our study results are in a good agreement with that reported by ROLARR group in terms of perioperative and pathological outcomes; however, this study has various limitations such as its retrospective nature, lack of data on bladder and sexual function, which may have reflected the quality of rectal dissection.

## Conclusion

In conclusion, perioperative and pathologic outcomes following robotic rectal resection reflect that it is safe, effective and reproducible by a minimally invasive novice surgeon. Careful patient selection, choosing less complex procedures and standardised protocol during the initial cases is of fundamental importance for a surgeon making a transition from open to robotic approach. Future studies are required to determine long-term oncologic and functional outcomes.
